# Change in mean salt intake over time using 24-h urine versus overnight and spot urine samples: a systematic review and meta-analysis

**DOI:** 10.1186/s12937-020-00651-8

**Published:** 2020-12-06

**Authors:** Joseph Alvin Santos, Ka Chun Li, Liping Huang, Rachael Mclean, Kristina Petersen, Gian Luca Di Tanna, Jacqui Webster

**Affiliations:** 1 The George Institute for Global Health, University of New South Wales, Sydney, NSW 2052 Australia; 2 Department of Preventive and Social Medicine, University of Otago, Dunedin, New Zealand; 3 Department of Nutritional Sciences, The Pennsylvania State University, University Park, PA USA

**Keywords:** Salt intake, 24-h urine, Overnight urine, Spot urine

## Abstract

**Background:**

Little is known about the capacity of overnight and spot urine samples to estimate changes in mean salt intake over time. The objective of this review was to compare the estimates of change in mean population salt intake based on 24-h urine and overnight/spot urine samples.

**Methods:**

Studies were systematically identified through searches of peer-reviewed databases (Medline, Embase, Global Health, Cochrane Central Register of Controlled Trials, and Cochrane Database of Systematic Reviews) and grey literature. Studies that reported estimates of mean salt intake for at least two time points based on both 24-h and overnight/spot urines were deemed eligible. The capacity of overnight/spot urine samples to estimate the change in mean salt intake was assessed both at the individual-study level and overall through random-effects meta-analyses. The level of heterogeneity was assessed through the I^2^ statistic. Subgroup and sensitivity analyses were conducted to explore possible sources of heterogeneity, and check the robustness of the findings from the primary analysis.

**Results:**

A total of 1244 records were identified, 50 were assessed as full text, and 14 studies met the criteria, capturing data on 7291 participants from seven countries. Nine and five studies collected overnight and spot urines, respectively. The comparison of the change in mean salt intake between 24-h and overnight/spot urines showed some inconsistencies at the individual study-level. The pooled mean change in salt intake was − 0.43 g/day (95% CI − 1.16 to 0.30; I^2^ = 95%) using 24-h urines, and − 0.22 g/day (− 0.65 to 0.20; I^2^ = 87%) using overnight/spot urines, with a pooled difference-in-differences between the two methods of 0.27 g/day (− 0.23 to 0.77; I^2^ = 89%). Subgroup analyses showed substantial heterogeneity for most subgroups. Sensitivity analyses did not change the effect observed in the primary analysis.

**Conclusion:**

The evidence for the capacity of overnight/spot urines to estimate changes in mean salt intake over time is uncertain. More research where overnight/spot urines are collected in parallel with 24-h urines is needed to enable a more in-depth evaluation of these alternative approaches to estimating change in mean salt intake.

**Supplementary Information:**

The online version contains supplementary material available at 10.1186/s12937-020-00651-8.

## Background

In 2013, the World Health Organization (WHO) recommended a 30% reduction in population salt intake as one of the global targets to reduce premature mortality from noncommunicable diseases (NCDs) by 25% by 2025 [1]. The recommended daily salt intake target is < 5 g for adults (equivalent to 2 g/day of sodium). In 2010, it was estimated that the global mean salt intake was 10 g/day, with more than 95% (181 of 187) of WHO Member States exceeding recommended limits [2]. Therefore, excessive salt intake is a worldwide public health problem.

Essential to achieving the WHO recommendation is establishing accurate benchmark salt intake levels and monitoring population salt intake regularly [3]. Currently, the gold standard method for measuring salt intake in an individual or population is 24-h urine collection, since most salt (about 90%) consumed in the previous 24 h is excreted in the urine in the form of sodium [4]. The major advantages of this approach include the objective nature of the measurement, and its ability to be applied across populations in a range of settings in a consistent manner. However, its limitations include the high burden imposed on the participants due to the complex nature of collection, which frequently leads to low participant rates and inaccurate urine collections [5, 6]. Furthermore, this method entails additional costs since participants must be provided with proper equipment such as urine bottles and collecting jugs, in addition to personnel costs associated with longer data collection period. For these reasons, its application is often limited in large population surveys.

Finding alternative methods for estimating salt intake has been the subject of much research in the past. Recent systematic reviews comparing dietary assessment methods (i.e. 24-h dietary recall, diet records, and food frequency questionnaire) with 24-h urine collections show that these approaches are inadequate for accurately estimating individual-level or population-level salt intake [7–9]. On the contrary, a systematic review assessing the capacity of spot urines, using 24-h urine as the reference method, showed that while this approach is inadequate for estimating individual-level salt intake, it can provide reasonable estimates of mean population salt intake [10]. The review found comparable mean population salt intake (9.3 g/day and 9.0 g/day from 24-h and spot urines, respectively), and excellent sensitivity and specificity at classifying mean salt intake as above or below the WHO recommended limit [10]. However, the study also showed the presence of proportional bias, i.e. spot urine samples overestimate salt intake when actual salt intake (based on 24-h urine) is lower, and underestimate salt intake when actual salt intake is higher [10]. This raises concerns about the applicability of using spot urines to measure changes in population salt intake over time, and whether this approach can deliver the same level of accuracy as that obtained from single time-point analyses [11]. Others have explored the use of timed overnight urine collection, which may yield more accurate estimates than spot urine due to the relatively long-term collection period [12, 13]. To our knowledge, whether overnight and spot urine samples can be used to measure changes in salt intake over time has not been systematically examined. The aim of this review was therefore to determine the capacity of overnight and spot urine samples to estimate changes in salt intake, compared to 24-h urines.

## Methods

### Databases and search terms

The *Preferred Reporting Items for Systematic Reviews and Meta-Analyses* (PRISMA) checklist guided the conduct of this review. A search for peer-reviewed literature was conducted using Medline, Embase, Global Health, Cochrane Central Register of Controlled Trials, and Cochrane Database of Systematic Reviews from their start date to September 2019. The search was not limited by date of publication or by language. Additional file 1 lists the search strategy in Medline, which was adapted for the other databases. The same search terms were used in Google Scholar, governmental and non-governmental websites to look for relevant grey literature. References of included studies were reviewed for further sources of information.

### Study selection

Search results were imported to EndNote X9 (Clarivate Analytics, 2019). All titles and abstracts were screened by two review authors (JS and KL), and potentially relevant articles were obtained in full text, and further assessed for eligibility. Any disagreement during the screening process was resolved through discussion. In order for a study to be eligible for inclusion, salt intake has to be measured using 24-h urines as the reference method, in addition to the criteria specified below, to allow for assessment of the applicability of using overnight or spot urine samples in measuring changes in salt intake over time.

- *Type of studies.* Randomised controlled trials (RCTs), controlled and uncontrolled pre-post studies, time-series studies, and repeated cross-sectional studies.
- *Type of participants.* General population, high risk groups, and population subgroups of any age and living in any region worldwide.
- *Type of outcome measures.* Studies were included if the primary or secondary outcomes provided information related to changes in mean salt intake measured using overnight/spot urine samples. Included studies monitored salt intake over time, evaluated salt intake as a response to an intervention, or compared salt intake estimates from overnight/spot urine and 24-h urine samples in at least two time points. Studies from which salt intake estimates could not be calculated (e.g. those that only reported average intake for multiple collections over the study duration, did not report values for baseline and follow-up, or only reported correlation coefficients) were excluded from the review.

### Data extraction and analysis

A data extraction form was developed for the purpose of this review. Data on study year, country of study, study design, type of participants, sample size, number of 24-h and overnight/spot urine samples collected, salt intake estimates from both methods, equations used for estimating salt intake from spot urines (where applicable), and length of follow-up were extracted. All sodium estimates were converted to salt intake in g/day for consistency using the following conversion factors: 1 mmol sodium = 23 mg sodium; 1 mg sodium = 2.54 mg sodium chloride or salt [14].

Given that the salt intake estimates based on overnight/spot urines were derived and reported in different ways across the studies, certain procedures were established to ensure that the data extraction was consistent, and the pooled analyses included data from each study only once. The following measures guided the data extraction and analyses:

- For studies where salt intake was measured for more than two time points [15–21], only the first and last measurements were included in the main analysis. Alternative follow-up data points (second-to-last) were used in the sensitivity analyses.
- For studies that used multiple spot-based equations [11, 20, 22–24], the equation considered by the authors as the primary analysis was included; however, if this was not specified, the equation that produced the best estimate (i.e. closest estimate to the 24-h urine in terms of absolute change in salt intake) was used. A sensitivity analysis using a single equation for all studies that used spot-based equations was carried out. The Intersalt equation was chosen given that it has been applied to different populations to estimate daily salt intake [10].
- For studies where no equation was used and sodium excretion was reported as a rate (e.g sodium excretion over 8 or 12 h) [15–18, 21, 25, 26], daily sodium excretion was obtained by inflating the values to a 24-h equivalent.

The capacity of overnight/spot urine samples to accurately determine the magnitude and direction of change at the individual study level was assessed by calculating the difference in mean salt intake over time (based on both 24-h and overnight/spot urines) using the equations outlined in the Cochrane Handbook for Systematic Review of Interventions [27]. For matched studies (i.e. same set of participants at baseline and follow-up), the within-subject correlation between baseline and follow-up measurements was obtained from each study, and was considered in the calculation of standard deviation (SD) and standard error (SE). If not available, the correlation was imputed as 0.487 for 24-h urines and 0.320 for overnight/spot urines (these were the median of *r* among the studies that reported this statistic). The difference-in-differences (i.e. change in salt intake estimated using overnight/spot urines minus the change in salt intake measured using 24-h urines) was also calculated per study, considering the correlation between 24-h and overnight/spot urines in the computation of SD and SE. For studies that did not report this correlation, the median of the correlations from the other studies (*r* = 0.459) was used. For studies with unequal sample sizes between the methods, a conservative approach was taken, by using the smaller sample size in the calculations.

The overall effect estimate was calculated as the mean difference (MD) with 95% confidence interval (CI), using the Sidik-Jonkman method for random-effects meta-analysis [28]. Pooled effect estimates using the method of DerSimonian and Laird [29] were also derived for comparative purposes. The main analysis combined the overnight and spot urine samples, but the difference between the two was explored in the subgroup analyses. Other factors explored in the subgroup analyses were the year of study; male-to-female sex ratio; median length of follow-up; median sample size; median salt intake at baseline based on 24-h urine; follow-up sample (*matched* vs *unmatched sample*), and; type of diet. The proportion of variability attributable to heterogeneity was assessed through calculating the I^2^ statistic. The direction and magnitude of the pooled mean differences based on 24-h urine and overnight/spot urines were compared. All analyses were conducted using Stata V16.0 for Windows (StataCorp, College Station, TX, USA) and RStudio (RStudio Inc., Boston MA, USA).

### Quality assessment

Quality of the included studies was assessed through a modified tool for evaluating dietary intake validation studies [30]. The tool uses five domains to rate the studies on a scale of 0 to 7, with the following interpretations: very good to excellent if the score was ≥5.0; good if the score was ≥3.5 and < 5.0; acceptable or reasonable if the score was ≥2.5 and < 3.5, and; poor if the score was < 2.5 (please see Additional file 2). For the purpose of this study, the *data collection* domain was modified to make it more relevant to 24-h and overnight/spot urine collection. Two authors (JS and KL) independently assessed the quality of the studies, and disagreements were resolved through discussion. A sensitivity analysis excluding studies of poor quality was conducted to check the robustness of the results in the main analysis.

## Results

### Search results and characteristics of studies

**Fig. 1:**
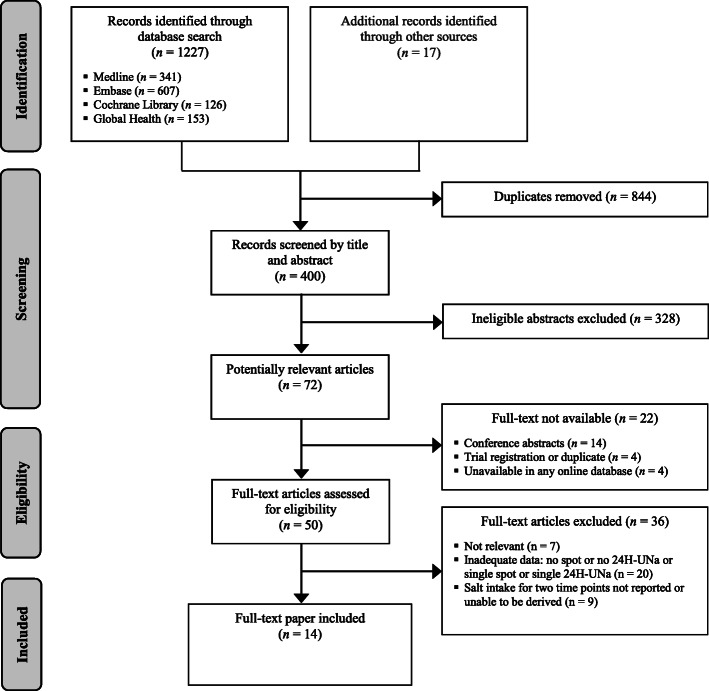
Flowchart of included studies

The search identified 1244 records, of which 72 were considered potentially eligible for full review (Fig. 1). Of these, 22 full-texts were unavailable (14 were conference abstract publications, four were trial registrations or duplicates, and four were unavailable in any online database). Thirty-six articles were excluded after full-text screening for the following reasons: inadequate (single) overnight/spot or 24-h urine data (*n* = 20), salt intake estimates were not reported or unable to be calculated based on the reported data (*n* = 9), and not relevant (*n* = 7). Ultimately, 14 studies were included in this review. The studies included 7291 participants from seven different countries reported between 1970 to 2019. There were five studies in China [16–18, 23, 24], three in the US [15, 25, 26], two in Japan [19, 31], and one study each in Viet Nam [22], Australia [11], South Korea [20], and Netherlands [21]. Sample sizes ranged from 20 to 2864. Participants’ age ranged from 10 to 75 years, with about equal men and women *(n* = 3579 and 3712, respectively) despite four studies only including men [15–18]. Eleven studies followed-up the same set of participants over time (matched samples), while the other three studies [11, 22, 23], which were community-based and also the largest studies in terms of sample size, used unmatched samples. Participants in three studies [15, 26, 31] were placed on a controlled dietary regimen (meals eaten were provided), while the rest were on their usual diet. Seven studies reported more than two data collection points. A summary of the characteristics of the included studies is provided in Table 1.

**Table 1:** Characteristics of studies and salt intake estimates based on 24-h and overnight/spot urine samples

First author, Year, Country	Mean age or range	Female (%)	Type of diet	Length of follow-up and type of sample	Equations used	SALT INTAKE ESTIMATES				
						Data point	24-h urine		Overnight/spot urine	
							N	Mean salt intake, g/day	N	Mean salt intake, g/day
**Studies that collected 24-h urine and overnight urine samples**										
Watson,1970 [25] *United States*	21–23 years	100	Usual diet	1 year; Matched sample	None; rate reported per hour	1 (B)^a^	25	5.96 ± 2.06	25	4.10 ± 1.64
						2 (Y1)	25	6.89 ± 2.46	25	5.58 ± 3.63
Luft, 1982 [26] *United States*	19–54 years	40	Controlled diet; meals in a clinical research center	15 days; Matched sample	None; rate reported per 8 h	1 (B)	43	7.89 ± 1.53	43	5.26 ± 2.30
						2 (D15)	43	8.12 ± 1.15	43	4.91 ± 1.15
Luft, 1982 [15] *United States*	19–32 years	0	Controlled diet; meals in a clinical research center	7 days; Matched sample	None; rate reported per 8 h	1 (B)	24	9.81 ± 6.30	24	5.24 ± 3.52
						2 (D2)	24	9.58 ± 4.01	24	5.59 ± 3.09
						3 (D3)	24	10.40 ± 2.58	24	5.61 ± 3.01
						4 (D4)	24	10.92 ± 2.58	24	5.43 ± 2.15
						5 (D5)	24	10.69 ± 2.86	24	5.63 ± 2.23
						6 (D6)	24	10.22 ± 2.86	24	5.19 ± 2.32
						7 (D7)	24	9.70 ± 2.58	24	5.26 ± 2.66
Liu, 1986 [16] *China*	30–50 years	0	Usual diet	3 months; Matched sample	None; rate reported per 12 h	1 (B)	49	13.51 ± 5.25	19	12.28 ± 7.12
						2 (NS)	49	14.58 ± 5.44	19	12.86 ± 7.24
						3 (NS)	49	15.34 ± 5.65	19	12.43 ± 5.34
						4 (NS)	49	13.82 ± 5.70	19	10.97 ± 5.11
						5 (NS)	49	14.81 ± 6.22	19	12.56 ± 7.08
						6 (M3)	49	13.81 ± 5.59	19	11.72 ± 6.20
Liu, 1987 [17] *China*	27–50 years Mean: 35	0	Usual diet	10 days; Matched sample	None; rate reported per 12 h	1 (B)	50	13.75 ± 5.71	50	13.27 ± 5.94
						2 (NS)	50	15.14 ± 5.71	50	13.82 ± 5.54
						3 (NS)	50	15.14 ± 6.48	50	14.27 ± 6.24
						4 (NS)	50	14.28 ± 5.19	50	12.76 ± 5.92
						5 (NS)	50	15.18 ± 6.48	50	13.81 ± 6.38
						6 (D10)	50	14.97 ± 4.91	50	13.80 ± 5.75
He, 1993 [18] *China*	19–55 years Mean: 37	0	Usual diet	3 days; Matched sample	None; rate reported per 8 h	1 (B)	63	8.58 ± 4.14	63	6.68 ± 4.01
						2 (D2)	63	9.08 ± 4.91	63	7.24 ± 4.89
						3 (D3)	63	9.65 ± 3.91	63	7.62 ± 4.36
Yasutake, 2015 [31], *Japan*	Mean: 22	55	Controlled diet	15 days; Matched sample	None; self-monitoring device used	1 (B)	10	8.60 ± 1.20	20	8.29 ± 0.98
						2 (D15)	12	4.40 ± 1.30	20	6.63 ± 1.44
Yasutake, 2016 [19], *Japan*	20–70 years Mean: 40	80	Usual diet	Varies: 2 weekdays and 1 holiday; Matched sample	None; self-monitoring device used	1 (B)	44	8.00 ± 1.80	50	8.00 ± 1.70
						2 (NS)	44	8.50 ± 2.60	50	8.10 ± 1.80
						3 (NS)	39	8.30 ± 3.30	50	8.30 ± 2.00
Kramers, 2019 [21]*, Netherlands*	18–70 years Mean: 46	52	Usual diet	6 weeks; Matched sample	None; rate reported per 8 h	1 (B)	27	9.29 ± 3.27	27	8.41 ± 3.79
						2 (W3)	27	8.70 ± 3.10	27	8.55 ± 3.51
						3 (W6)	27	8.88 ± 3.91	27	7.57 ± 4.49
**Studies that collected 24-h urine and spot urine samples**										
Do, 2016 [22] *Viet Nam*	25–64 years Mean: 45	53	Usual diet before and after an intervention	1 year; Unmatched sample	5 equations used; Intersalt treated as the main analysis	1 (B)	88	9.43 ± 3.69	509	8.48 ± 2.13
						2 (Y1)	73	7.44 ± 4.09	511	8.05 ± 2.11
Petersen, 2016 [11], *Australia*	18 years and up	56	Usual diet	3 years; Unmatched sample (with some matched)	6 equations used; Kawasaki provided the closest estimate of the change	1 (B)	1000	8.37 ± 3.43	1000	11.69 ± 3.45
						2 (Y3)	1012	7.89 ± 3.66	1012	11.27 ± 3.52
Huang, 2018 [23] *China*	60 years and up Mean: 66	50	Usual diet before and after an intervention	Varies: up to 24 months Unmatched sample (with some matched)	8 equations used; using *individual volume spot* provided the closest estimate of the change	1 (B)	532	10.92 ± 4.57	532	12.19 ± 7.37
						2 (Y2)	2864	9.91 ± 4.32	2864	10.67 ± 6.60
Dong, 2018 [24] *China*	10–15 years Mean: 12	48	Usual diet	2–3 weeks; Matched sample	8 equations used; Whitton provided the closest estimate of the change	1 (B)	284	6.56 ± 2.69	284	9.92 ± 2.50
						2 (W3)	284	7.26 ± 2.91	284	10.17 ± 2.48
Kim, 2018 [20] *South Korea*	19–75 years Mean: 50	49	Usual diet	16 weeks; Matched sample with two treatment groups	5 equations used; Intersalt provided the closest estimate of the change for both groups consistently	1 (B)	228	9.00 ± 4.07	228	7.41 ± 2.21
						2 (M2)	228	9.15 ± 4.12	228	7.61 ± 2.35
						3A (M4)	118	8.48 ± 3.51	118	7.62 ± 2.16
						3B (M4)	111	7.12 ± 3.48	111	7.03 ± 2.19

^a^ *B* baseline; *D* day; *W* week; *M* month; *Y* year; *NS* not specified

In terms of quality, seven studies were rated as of *reasonable* quality, four were of *good* quality, and three were of *poor* quality. None of the studies was rated as *excellent* quality (Table 2).

**Table 2:** Quality assessment of the included studies [30]

Domain	Specific item	Watson 1970 [25]	Luft 1982 [26]	Luft 1982 [15]	Liu 1986 [16]	Liu 1987 [17]	He 1993 [18]	Yasutake 2015 [31]	Yasutake 2016 [19]	Do 2016 [22]	Petersen 2016 [11]	Huang 2018 [23]	Dong 2018 [24]	Kim 2018 [20]	Kramers 2019 [21]
**Sample and sample size**	Non-homogenous sample	0.0	0.5	0.0	0.0	0.0	0.5	0.5	0.5	0.5	0.5	0.5	0.0	0.5	0.5
	*N* > 50	0.0	0.0	0.0	0.0	0.0	0.5	0.0	0.0	0.5	0.5	0.5	0.5	0.5	0.0
**Statistics to assess validity**	Test of mean, median or difference	1.0	1.0	1.0	1.0	1.0	1.0	1.0	1.0	1.0	1.0	1.0	1.0	1.0	1.0
	Correlations	0.5	0.0	0.5	0.5	0.5	0.5	0.5	0.5	0.0	0.0	0.0	1.5	0.5	0.0
	Agreement	0.0	0.0	0.5	0.0	0.5	0.0	0.5	0.5	0.0	0.5	0.0	0.5	0.5	0.0
**Data collection**	Verbal or written instructions to collect urine	0.5	0.5	0.5	0.5	0.5	0.5	0.0	0.5	0.5	0.5	0.5	0.5	0.5	0.5
	Spillage or missed voids assessed post-collection	0.0	0.0	0.0	0.5	0.5	0.5	0.0	0.0	0.5	0.5	0.5	0.5	0.0	0.0
**Seasonality**	Considered	0.0	0.0	0.0	0.0	0.0	0.0	0.0	0.0	0.0	0.0	0.0	0.0	0.0	0.0
**Supplements**	Included and data considered in analysis	0.0	0.0	0.0	0.0	0.0	0.0	0.0	0.0	0.0	0.0	0.0	0.0	0.0	0.0
**SCORE**^**a**^		**2.0**	**2.0**	**2.5**	**2.5**	**3.0**	**3.5**	**2.5**	**3.0**	**3.0**	**3.5**	**3.0**	**4.5**	**3.5**	**2.0**
**QUALITY**^**b**^		**P**	**P**	**A**	**A**	**A**	**G**	**A**	**A**	**A**	**G**	**A**	**G**	**G**	**P**

^a^ Score interpretations: ≥5.0, very good to excellent quality; ≥3.5 and < 5.0, good quality; ≥2.5 and < 3.5, acceptable or reasonable quality; < 2.5, poor quality

^b^ *P* poor; *A* acceptable; *G* good

### Characteristics of urine samples collected

Of the 14 studies included in this review, nine collected overnight urine samples, seven of which reported the rate of sodium excretion [15–18, 21, 25, 26], while two studies used a self-monitoring device to estimate daily salt intake [19, 31]. On the other hand, five studies collected spot urine samples, and utilised different spot-based equations to estimate daily salt intake [11, 20, 22–24]. The interval between salt intake measurements was short (days or weeks), except for four studies that collected the second salt intake estimate after at least a year [11, 22, 23, 25]. The duration of follow-up ranged from 3 days to 3 years. In 12 of the 14 studies, overnight/spot urine was collected as part of the 24-h urine.

### Change in salt intake at the individual study level

**Fig. 2:**
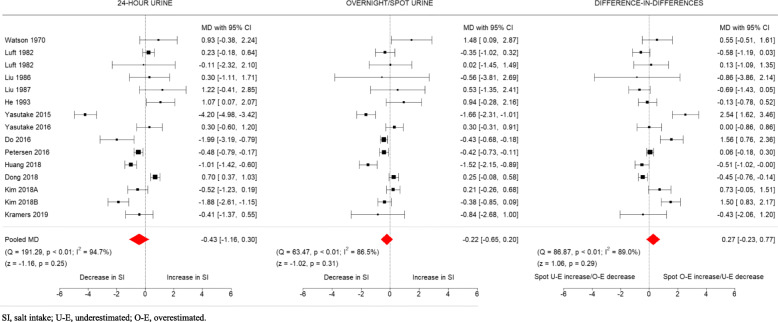
Change in salt intake over time based on 24-h and overnight/spot urine samples

Based on 24-h urines, five studies [11, 20, 22, 23, 31] reported a reduction in salt intake over time, and two [18, 24] an increase (Fig. 2). Overnight/spot urines were able to detect the reductions in four of the five studies, but unable to detect the decrease in one, and the increase in salt intake in the two studies. In one study [25], 24-h urines showed no change, but overnight urines showed an increase. The difference-in-differences analysis showed that overnight/spot urines underestimated the decrease in salt intake shown in three studies, and underestimated the increase in salt intake in one study.

### Pooled change in salt intake using 24-h and overnight/spot urine samples

**Fig. 3:**
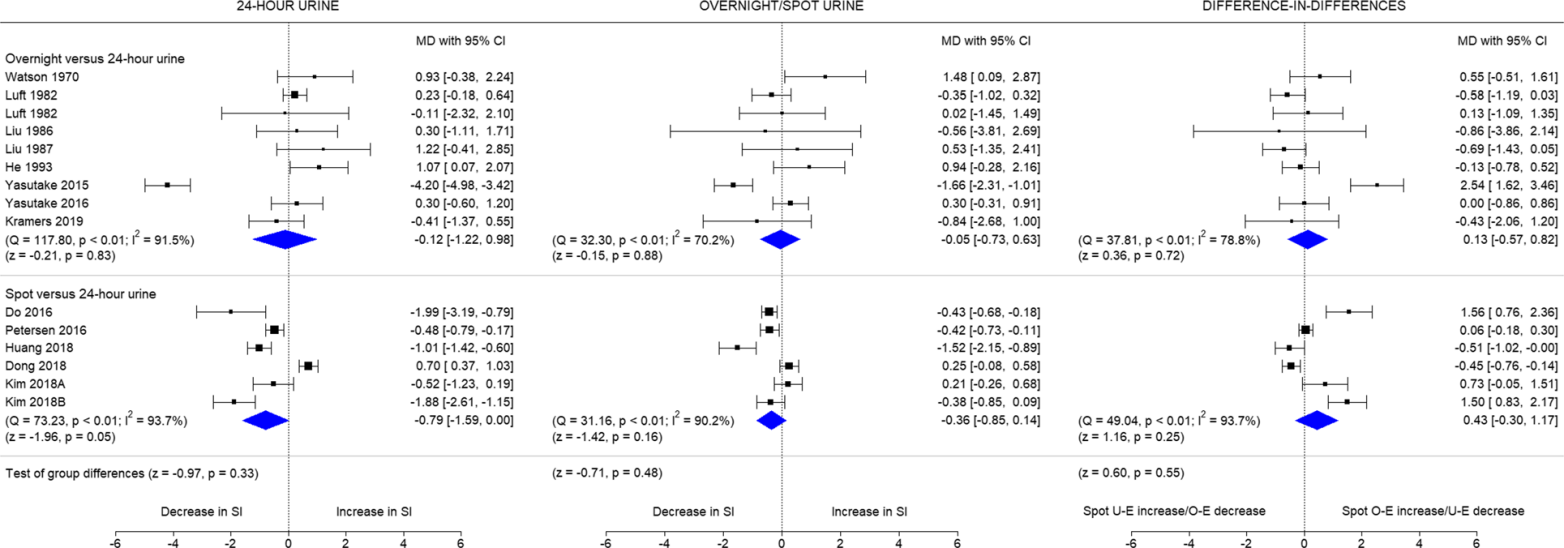
Change in salt intake over time by type of urine samples compared

The overall change in salt intake over time based on 24-h urines was − 0.43 g/day (95% CI − 1.16 to 0.30), while the change based on overnight/spot urine samples was − 0.22 g/day (95% CI − 0.65 to 0.20) (Fig. 2). The pooled effect estimates using the method of DerSimonian and Laird are shown in Additional file 3**.** The level of variability due to heterogeneity was substantial for both methods (I^2^ = 95 and 87% for 24-h and overnight/spot urines, respectively). The pooled difference-in-differences was 0.27 g/day (95% CI − 0.23 to 0.77; I^2^ = 89%). Separate analyses of overnight and spot urines (Fig. 3) showed absence of group differences between the two methods, with the results similar to the main analysis where they were combined.

**Fig. 4:**
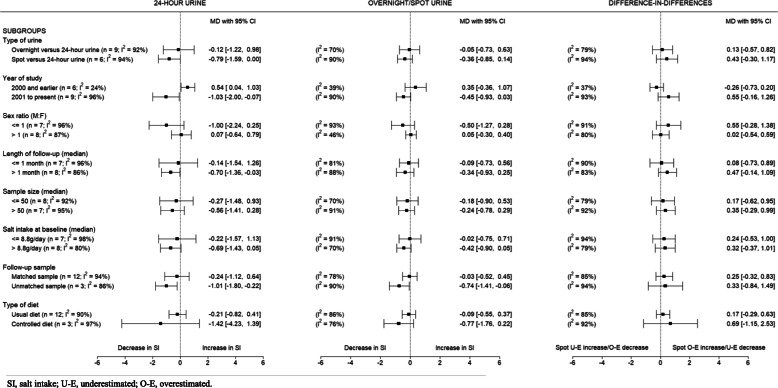
Change in salt intake over time based on 24-h and overnight/spot urine samples by subgroups

The results of the eight subgroup analyses conducted are summarised in Fig. 4. The forest plot for each subgroup is shown in Additional file 4. For most subgroups, the level of variability due to heterogeneity was substantial; and only a few subgroups had less heterogeneity compared to the main analysis. There were no major subgroup differences found, apart from the comparison by the year of study (i.e. year 2000 and earlier versus year 2001 to present) using 24-h urines. For all subgroups, the direction of change (positive or negative) was the same for 24-h and overnight/spot urines. However, the magnitude of change measured by overnight/spot urines was *always* smaller compared to 24-h urine.

**Fig. 5:**
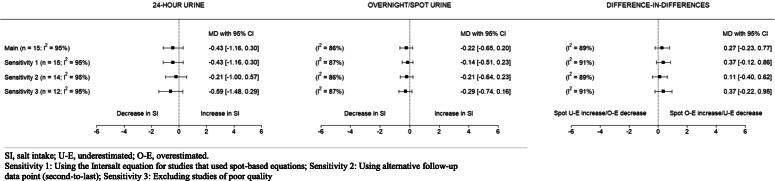
Change in salt intake over time based on 24-h and overnight/spot urine samples: Sensitivity analyses

The results of the three sensitivity analyses, namely using the Intersalt equation for studies that used spot-based equations, using alternative follow-up data points, and excluding studies of poor quality, are presented in Fig. 5. The pooled mean differences (and their confidence intervals) were similar across the sensitivity analyses, showing no change in mean salt intake over time. The forest plot for each sensitivity analysis is shown in Additional file 5.

## Discussion

This review identified 14 studies that measured and reported mean salt intake for at least two time points using both 24-h and overnight/spot urines. At the *individual-study level,* the comparison of the change in mean salt intake showed some inconsistencies in terms of magnitude and direction, although the difference-in-differences analysis suggests that overnight/spot urines tend to underestimate either the decrease or increase in mean salt intake, compared to 24-h urines. In the *pooled analyses,* both methods showed no change in mean salt intake over time; nonetheless, direct comparison of the pooled effect estimates generated from 24-h and overnight/spot urines showed that the direction of change (positive or negative) was the same between the two methods, although the magnitude of change generated from overnight/spot urines was less than the change detected by 24-h urines. This observation was consistent across the main analysis, the eight subgroup analyses, and three sensitivity analyses. However, it should be interpreted in light of the substantial heterogeneity found, and the variability in the quality of the included studies. We explored possible sources of heterogeneity through subgroup analyses, and while some subgroups showed less within-subgroup variability, this did not fully address the between-study variability found in the main analysis, precluding us from drawing any firm conclusions regarding the capacity of overnight/spot urines to estimate the change in salt intake over time.

The tendency of spot urines to underestimate the change in mean salt intake has been highlighted in a previous systematic review and meta-analysis that compared estimates of mean salt intake from 24-h urine and spot urines at one time point [10]. In our pooled analyses, the magnitude of change underestimated by overnight/spot urines was about 60%, and ranged from 29 to 109% (median 56%) in the subgroup analyses. This is likely the result of proportional bias, as established in previous studies [10]. It is important to investigate this further, since if spot and overnight urines truly and consistently underestimate the change in mean salt intake over time, then mathematical adjustments might be possible, and, any change generated from overnight/spot urines could be treated as the minimum effect that could be expected from an intervention.

At the individual study level, overnight/spot urine samples did not consistently detect the change in salt intake. Reductions were detected in five studies based on 24-h urines [11, 20, 22, 23, 31]. For these, three were underestimated by spot urines by 13 to 78%, one was overestimated by 50%, and one was undetected. Two studies [18, 24] showed an increase in mean salt intake using 24-h urines that was not detected by overnight/spot urines. It should be noted that in the paper by He et al. [18], the changes in salt intake estimated using 24-h and overnight urines were relatively close (1.07 g/day and 0.94 g/day, respectively), but the change shown by overnight urines was not *statistically significant*. These differences in detecting statistically significant changes might be related to the variability (SD) of the estimates obtained from overnight/spot urine samples. It appears that using the equations derived from regression analysis to convert spot urine sodium concentration to 24-h excretion estimates leads to a lower SD of the salt intake estimates [11, 20, 22–24]. This is *counterintuitive*, given that spot urines only measure salt intake at one time point (during the day), so they are expected to show higher variability. Petersen et al. [11] suggested that the lower variability is potentially due to the inclusion of other factors such as age, sex, weight and BMI in these spot-based equations, as these factors are less variable and unlikely to be affected by the change in salt intake. Interestingly, the use of overnight urines was not shown to be more effective at detecting change in salt intake than spot urines compared with 24-h urine collection. This implies that the extra participant and research burden of collection of overnight urines does not confer any advantage over spot urine collection, and remains inferior to 24-h urine collection for estimating change in salt intake.

Furthermore, our results suggest that determining the spot-based equation that best predicts mean salt intake *at one time point*, then using the same equation to monitor change in salt intake may lead to biased conclusions. In the Australian study [11], for example, the Intersalt and Toft equations produced reasonable estimates of salt intake at each period of collection, yet, the estimates of change were not as good as that generated by the Kawasaki equation, despite it substantially overestimating salt intake levels at each time point. This was also the case for the study in Viet Nam [22], where the Intersalt and Tanaka equations underestimated the change to a greater extent compared to the other spot equations, even though they generated the closest salt intake estimates at baseline and follow-up. These results suggest that the capacity of one spot equation to (1) predict salt intake and (2) to assess the change in salt intake over time might be different, even when applied in the same population; hence, it might be necessary to identify and validate which equation to use for what purpose. In the SHAKE Technical Package for Salt Reduction [3], the WHO suggests that spot urine samples may be used to obtain an estimate of mean population salt intake in countries where 24-h urine collection is not feasible, after baseline validation measures have been conducted. However, if countries intend to use spot urines to monitor changes in salt intake, then further research is needed to better understand the appropriateness of the different spot-based equations for that purpose. Thus, it is essential for countries to still collect 24-h urine samples from a subsample of the population when conducting surveys based on spot urine samples, to allow calibration of estimates using different equations.

A key strength of this review is that it included all study types, population groups and grey literature, and the search was not limited by publication date or language. The large number of studies allowed us to conduct subgroup analyses to explore possible sources of heterogeneity as well as to examine the capacity of overnight/spot urines in measuring change in salt intake in different subgroups. Our sensitivity analyses showed the robustness of the findings based on the main analysis. A key limitation is that in most studies, overnight/spot urine was collected as part of 24-h urine collection, so it is possible that these analyses overestimate the capacity of spot/overnight urines to detect change relative to 24-h urines [32]. In addition, none of the studies assessed the completeness of 24-h urines using para-aminobenzoic acid–the gold standard method for assessing urine completeness [33], so it is possible that the 24-h salt excretion was underestimated.

## Conclusion

In summary, the data presented here suggest that the capacity of overnight/spot urine samples to measure changes in salt intake over time needs further investigation. The studies identified to date are heterogeneous and only from a handful of countries. Additional well-designed and adequately powered studies where overnight/spot urines are collected in parallel with 24-h urines are needed to enable a more in-depth quantitative assessment of the applicability of overnight/spot urine samples to measure the change in mean salt intake, taking into account the various factors that may affect salt intake estimates.

## Supplementary Information

**Additional file 1.** Full search strategy in Medline. Additional file 1 contains the full search strategy (search terms and syntax) used in the Medline database.

**Additional file 2.** Quality assessment tool. Additional file 2 contains the adapted tool for evaluating the quality of dietary intake validation studies.

**Additional file 3.** Random effects model based on the DerSimonian-Laird method. Additional file 3 contains the pooled effect estimates using the method of DerSimonian and Laird.

**Additional file 4.** Subgroup analyses forest plots. Additional file 4 contains the forest plots of the subgroup analyses conducted: (a) by year of study; (b) by male-to-female sex ratio; (c) by median length of follow-up; (d) by median sample size; (e) by median salt intake at baseline based on 24-h urine; (f) by follow-up sample, and; (g) by type of diet.

**Additional file 5.** Sensitivity analyses forest plots. Additional file 5 contains the forest plots of the three sensitivity analyses conducted: (a) using the Intersalt equation for studies that used spot-based equation; (b) using alternative follow-up data point (second-to-last), and; (c) excluding studies of poor quality.

**Additional file 6.** PRISMA 2009 Checklist. Additional file 6 contains the completed PRISMA checklist.

## Data Availability

All data generated or analysed during this study are included within the article and additional files. The contents of this manuscript are based on a Master’s thesis (2017) submitted to the School of Public Health, Faculty of Medicine, The University of Sydney [34].

## Abbreviations

WHO: World Health Organization
NCD: Non-communicable disease
g/day: Grams per day
RCT: Randomised controlled trial
SD: Standard deviation
SE: Standard error
MD: Mean difference
CI: Confidence interval
