# Supplementary material for: Change in mean salt intake over time using 24-h urine versus overnight and spot urine samples: a systematic review and meta-analysis
Source: Nutr J. 2020 Dec 6;19:136. doi: 10.1186/s12937-020-00651-8 (PMC7720567; doi:10.1186/s12937-020-00651-8)
Supplement: Supplementary file 1 — Additional file 1. Full search strategy in Medline. Additional file 1 contains the full search strategy (search terms and syntax) used in the Medline database. [file 12937_2020_651_MOESM1_ESM.docx]

**Additional file 1.** Full search strategy in Medline

| **NO** | **SEARCHES** |
| --- | --- |
| 1 | ((24-h* or 24h* or 24 h*) adj5 urin*).tw. |
| 2 | ((spot or spot- or casual or random or timed or timed- or nocturnal or overnight or morning or fractional or afternoon or evening or sample) adj5 urin*).tw. |
| 3 | exp sodium/ |
| 4 | ((diet* or chloride or intake or excret* or ingest* or consump* or eat*) adj5 salt).tw. |
| 5 | ((diet* or chloride or intake or excret* or ingest* or consump* or eat*) adj5 sodium).tw. |
| 6 | ((diet* or chloride or intake or excret* or ingest* or consump* or eat*) adj5 Na).tw. |
| 7 | (change* or evaluat* or trend* or monitor* or valid* or increase or decrease or differen*).tw. |
| 8 | 1 and 2 |
| 9 | 3 or 4 or 5 or 6 |
| 10 | 7 and 8 and 9 |
