# Supplementary material for: Change in mean salt intake over time using 24-h urine versus overnight and spot urine samples: a systematic review and meta-analysis
Source: Nutr J. 2020 Dec 6;19:136. doi: 10.1186/s12937-020-00651-8 (PMC7720567; doi:10.1186/s12937-020-00651-8)
Supplement: Supplementary file 2 — Additional file 2. Quality assessment tool. Additional file 2 contains the adapted tool for evaluating the quality of dietary intake validation studies. [file 12937_2020_651_MOESM2_ESM.docx]

**Additional file 2.** Quality assessment tool

From Serra-Majem L, Frost Andersen L, Henríque-Sánchez P, Doreste-Alonso J, Sánchez-Villegas A, Ortiz-Andrelluchi A, et al. Evaluating the quality of dietary intake validation studies. The British Journal of Nutrition. 2009;102 Suppl 1:S3-9

| **VARIABLE** | **SPEFICIC VARIABLE** | **POINTS** |
| --- | --- | --- |
| **Sample and sample size**  (maximum 1 point) | - The sample was not homogenous for certain variables such as sex, socio-economic status, smoking and obesity | **0.5** |
|  | - The sample was of more than 50 individuals, when using biomarkers as the gold standard | **0.5** |
| **Statistics to assess validity**  (maximum 3 points) | - Comparison between the methods’ means, median or difference was conducted | **1.0** |
|  | - A measure of correlation was reported in the study (*select one with highest score*) - Correlation (crude) - Adjusted correlation (energy) - Deattenuated or intraclass correlation | **0.5**  **1.0**  **1.5** |
|  | - Statistics to assess agreement or misclassification were utilised (classification or Bland-Altman plot) | **0.5** |
| **Data collection**  (maximum of 1 point) | - Data were gathered by face to face interview   **NOTE:** Face to face interview is not relevant to 24-hour and overnight/spot urine collection; hence, this was scored based on: |  |
|  | - Whether verbal or written instructions to collect the urine samples were given to the participants | **0.5** |
|  | - Whether the participants were asked for spillage and missed voids post-collection | **0.5** |
| **Seasonality**  (maximum of 0.5 point) | - Seasonality was considered | **0.5** |
| **Supplements** | - Supplement intake (e.g Vitamin D, E, B, folic acid, etc) was included and data considered in analysis | **1.5** |

The tool uses five domains to rate the studies on a scale of 0 to 7, with the following interpretations:

- Very good/excellent: score ≥ 5.0
- Good: 3.5 ≤ score < 5
- Acceptable/reasonable: 2.5 ≤ score < 3.5
- Poor: score < 2.5

For the purpose of this study, the *data collection* domain was modified to make it more relevant to 24-hour and overnight/spot urine collection.
