# Supplementary material for: Change in mean salt intake over time using 24-h urine versus overnight and spot urine samples: a systematic review and meta-analysis
Source: Nutr J. 2020 Dec 6;19:136. doi: 10.1186/s12937-020-00651-8 (PMC7720567; doi:10.1186/s12937-020-00651-8)
Supplement: Supplementary file 3 — Additional file 3. Random effects model based on the DerSimonian-Laird method. Additional file 3 contains the pooled effect estimates using the method of DerSimonian and Laird. [file 12937_2020_651_MOESM3_ESM.docx]

**Additional file 3.** Random effects model based on the DerSimonian-Laird method


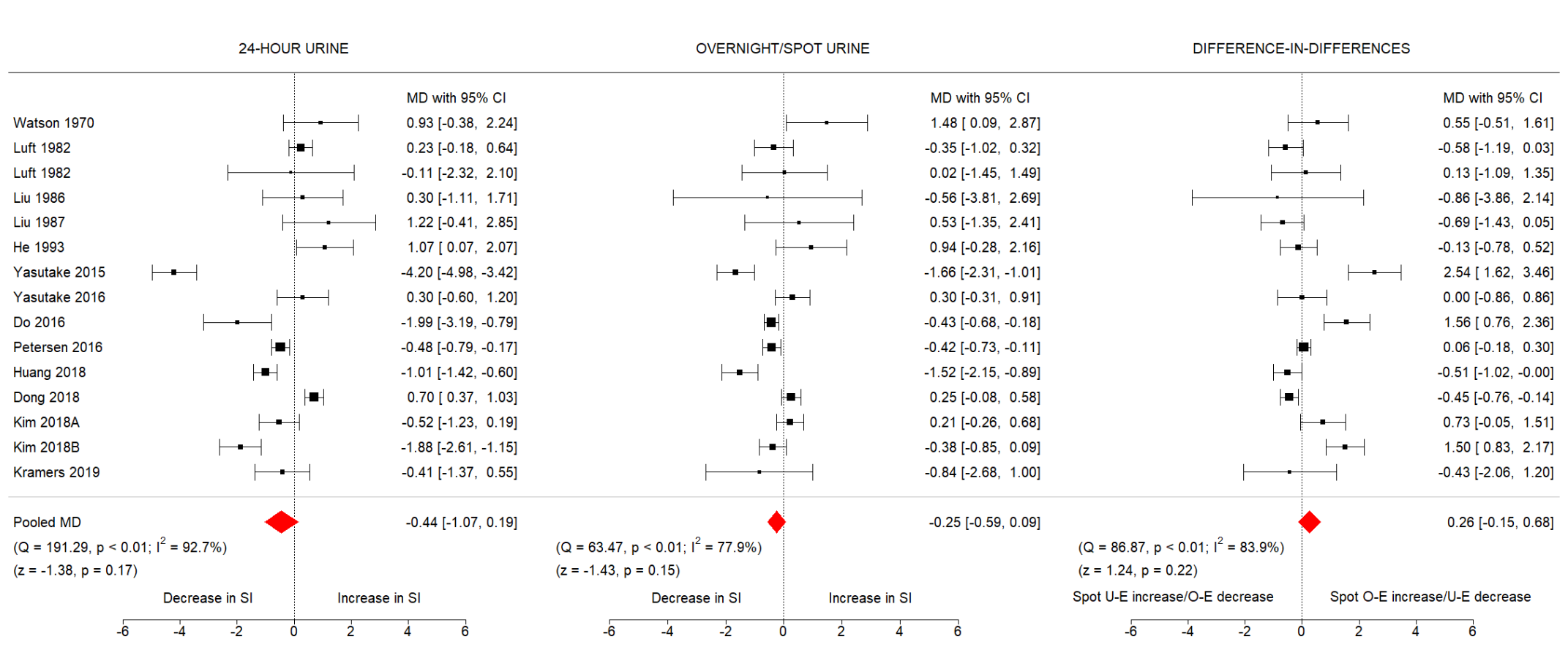


SI, salt intake; U-E, underestimated; O-E, overestimated.
