# Supplementary material for: Change in mean salt intake over time using 24-h urine versus overnight and spot urine samples: a systematic review and meta-analysis
Source: Nutr J. 2020 Dec 6;19:136. doi: 10.1186/s12937-020-00651-8 (PMC7720567; doi:10.1186/s12937-020-00651-8)
Supplement: Supplementary file 4 — Additional file 4. Subgroup analyses forest plots. Additional file 4 contains the forest plots of the subgroup analyses conducted: (a) by year of study; (b) by male-to-female sex ratio; (c) by median length of follow-up; (d) by median sample size; (e) by median salt intake at baseline based on 24-h urine; (f) by follow-up sample, and; (g) by type of diet. [file 12937_2020_651_MOESM4_ESM.docx]

**Additional file 4.** Subgroup analyses forest plots

**A. By year of study**


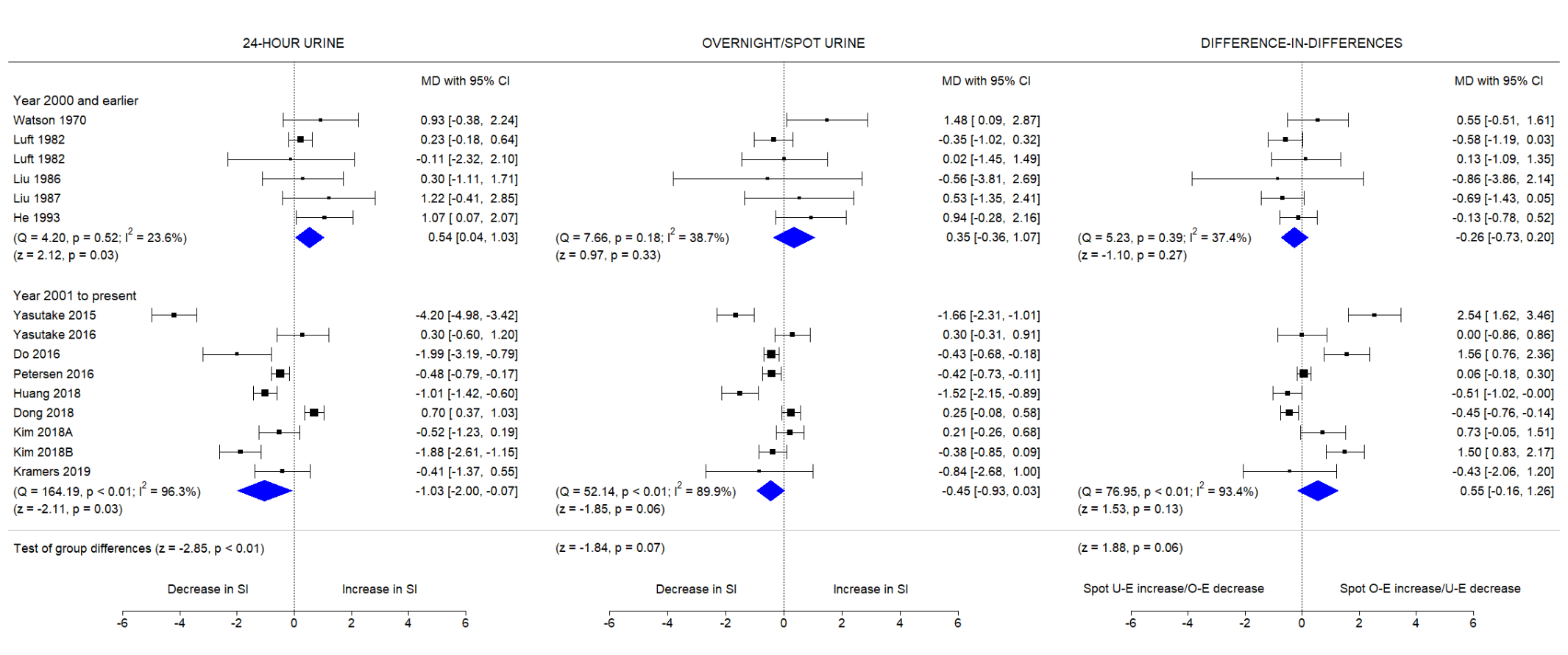


SI, salt intake; U-E, underestimated; O-E, overestimated.

**B. By male-to-female sex ratio**


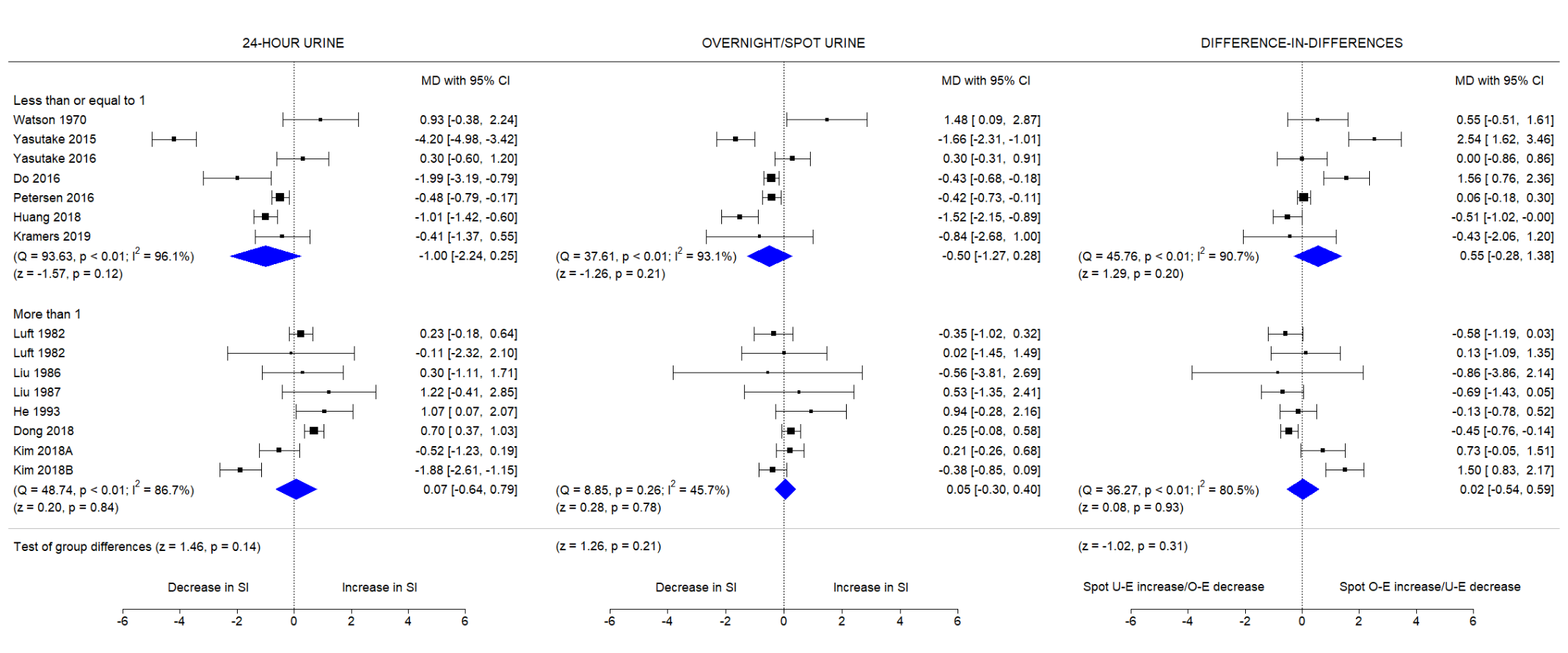


SI, salt intake; U-E, underestimated; O-E, overestimated.

**C. By length of follow-up (median)**


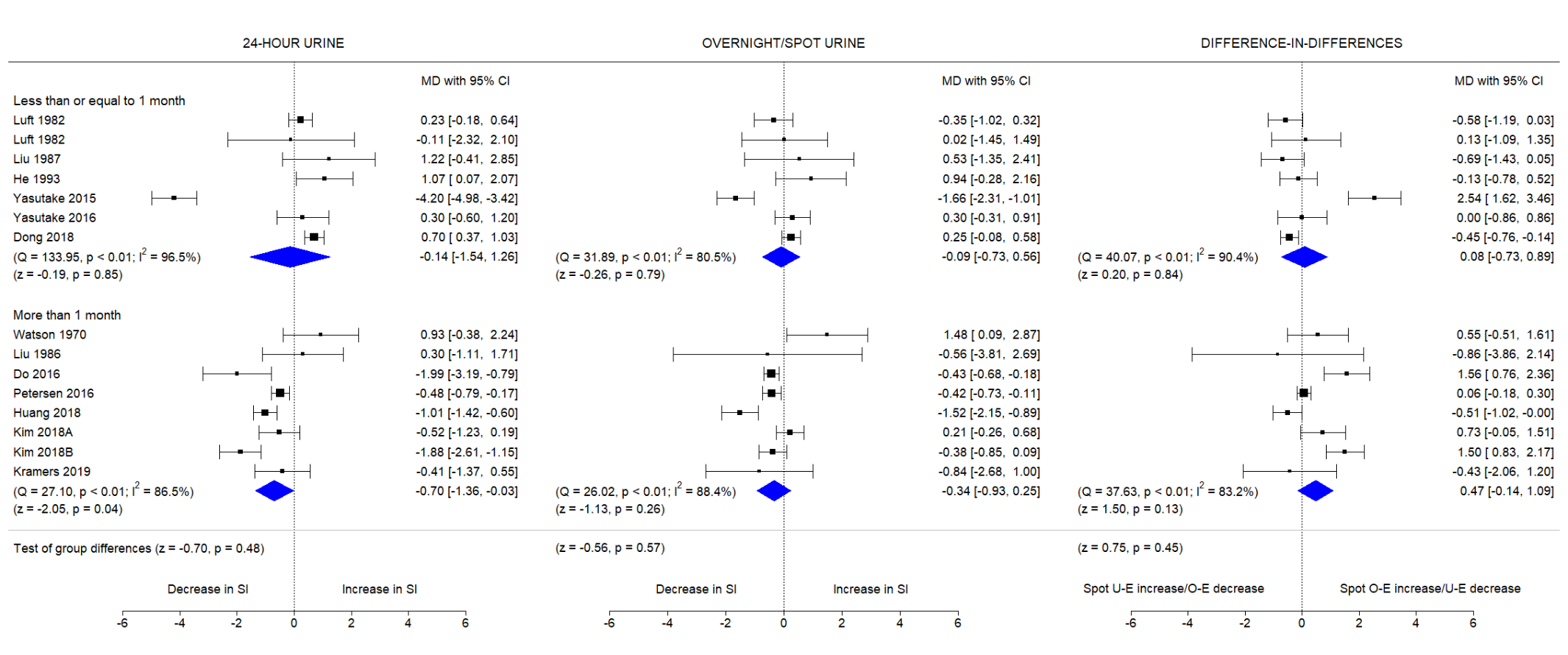


SI, salt intake; U-E, underestimated; O-E, overestimated.

**D. By sample size (median)**


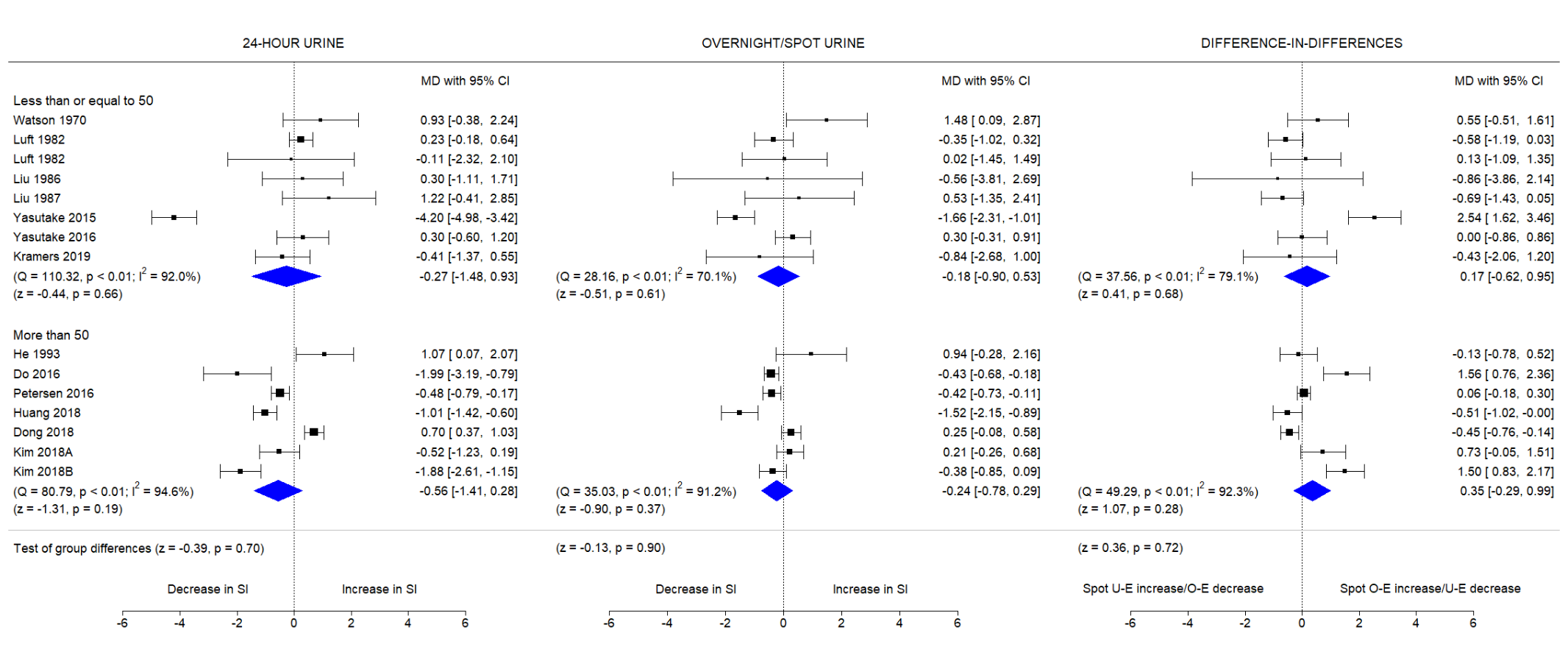


SI, salt intake; U-E, underestimated; O-E, overestimated.

**E. By salt intake at baseline based on 24-hour urine (median)**


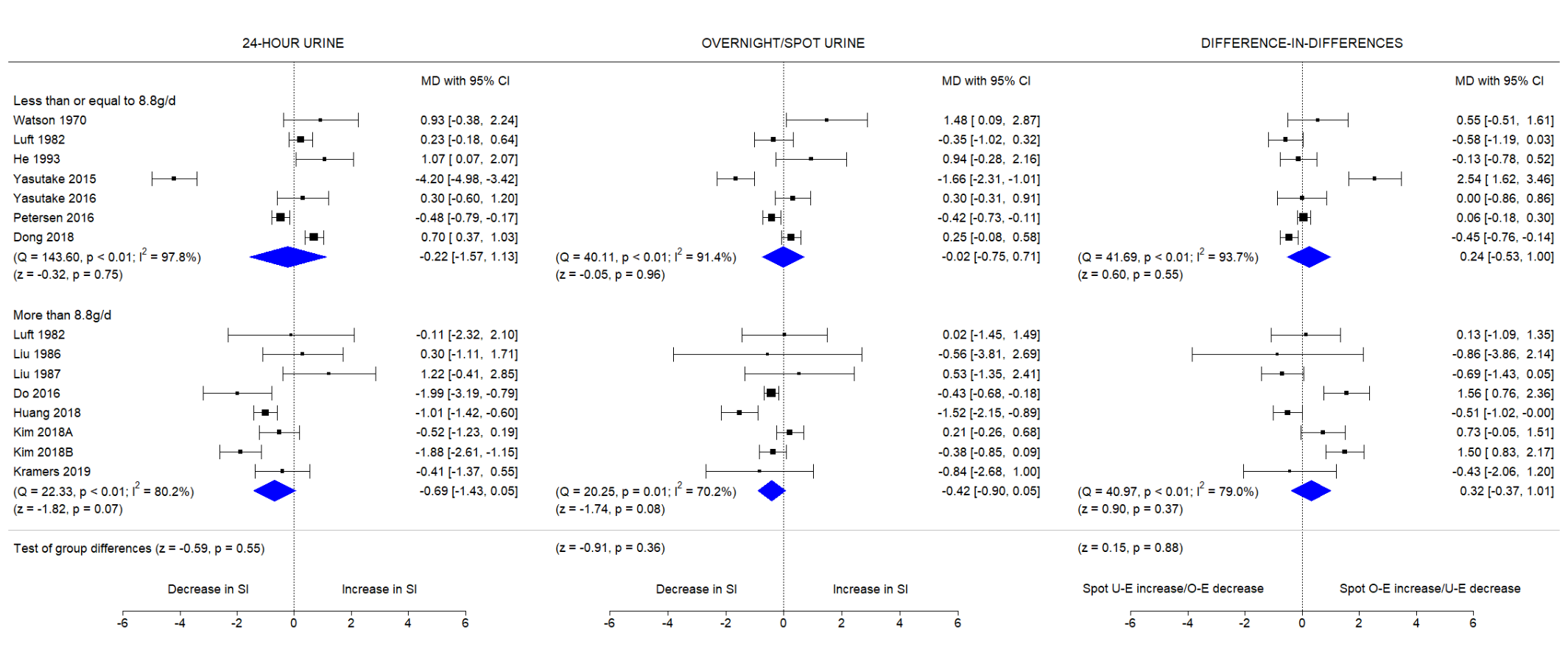


SI, salt intake; U-E, underestimated; O-E, overestimated.

**F. By follow-up sample**


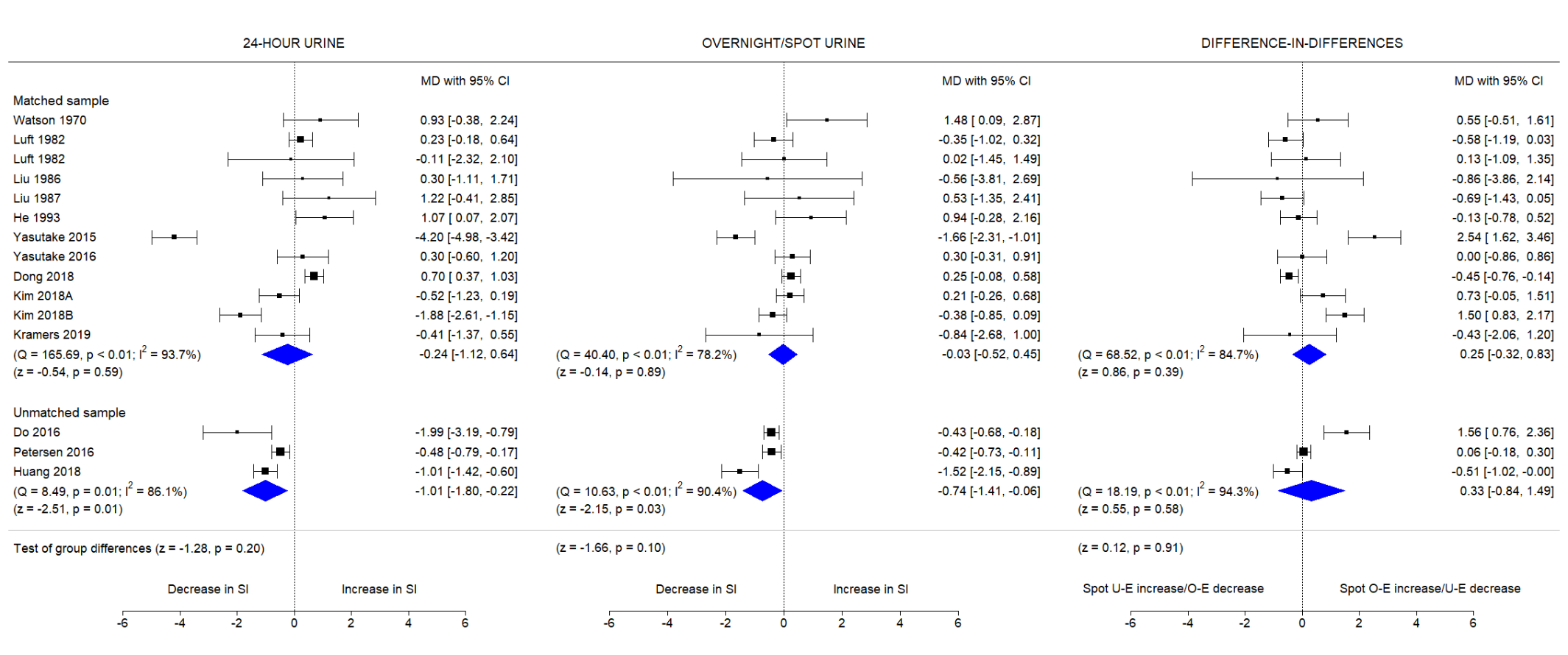


SI, salt intake; U-E, underestimated; O-E, overestimated.

**G. By type of diet**


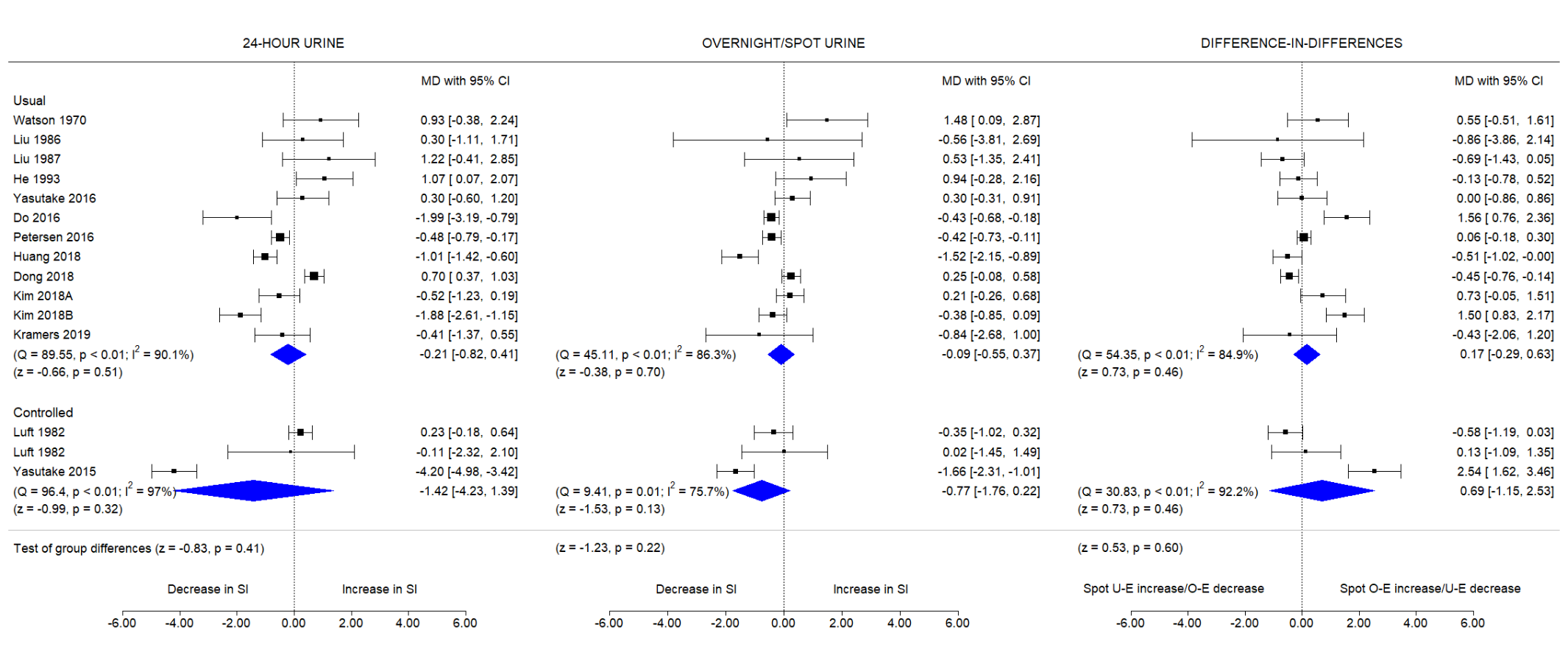


SI, salt intake; U-E, underestimated; O-E, overestimated.
