# Supplementary material for: Change in mean salt intake over time using 24-h urine versus overnight and spot urine samples: a systematic review and meta-analysis
Source: Nutr J. 2020 Dec 6;19:136. doi: 10.1186/s12937-020-00651-8 (PMC7720567; doi:10.1186/s12937-020-00651-8)
Supplement: Supplementary file 5 — Additional file 5. Sensitivity analyses forest plots. Additional file 5 contains the forest plots of the three sensitivity analyses conducted: (a) using the Intersalt equation for studies that used spot-based equation; (b) using alternative follow-up data point (second-to-last), and; (c) excluding studies of poor quality. [file 12937_2020_651_MOESM5_ESM.docx]

**Additional file 5.** Sensitivity analyses forest plots

**A. Using the Intersalt equation for studies that used spot-based equation**


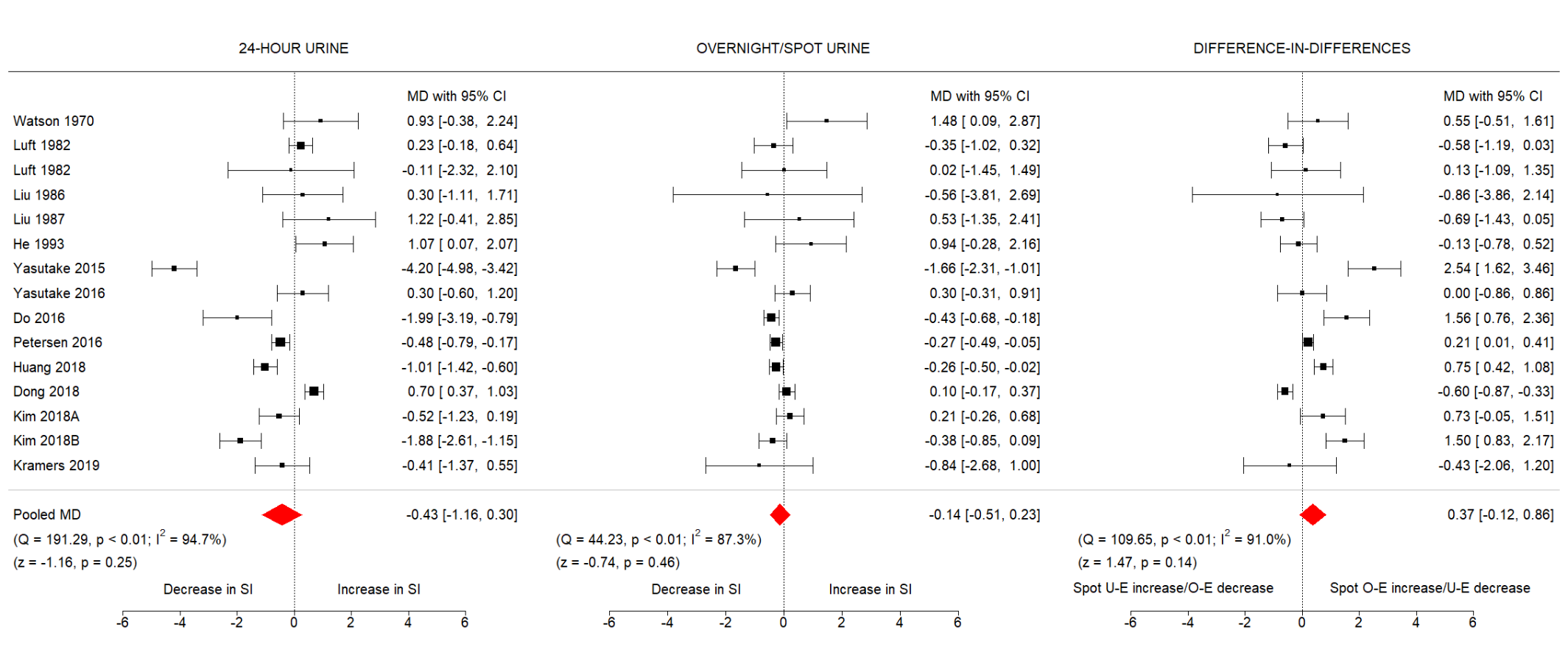


SI, salt intake; U-E, underestimated; O-E, overestimated.

**B. Using alternative follow-up data point (second-to-last)**


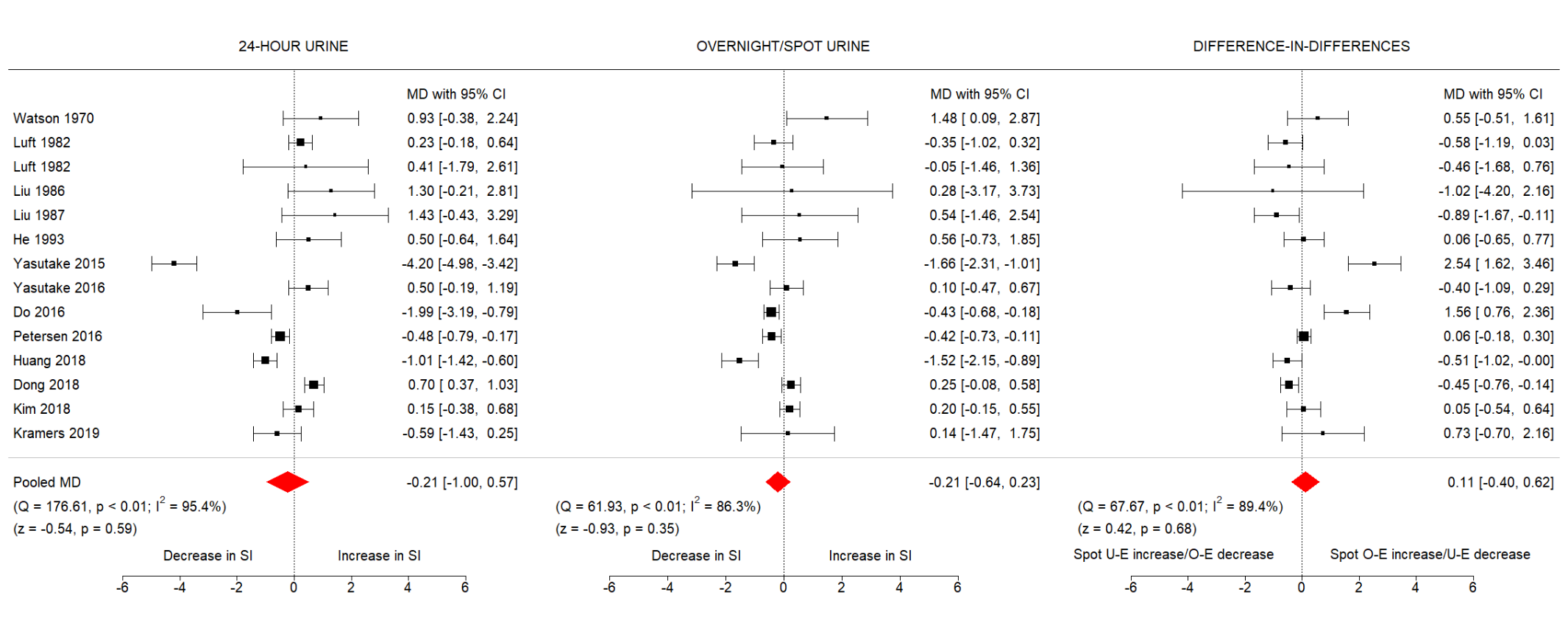


SI, salt intake; U-E, underestimated; O-E, overestimated.

**C. Excluding studies of poor quality**


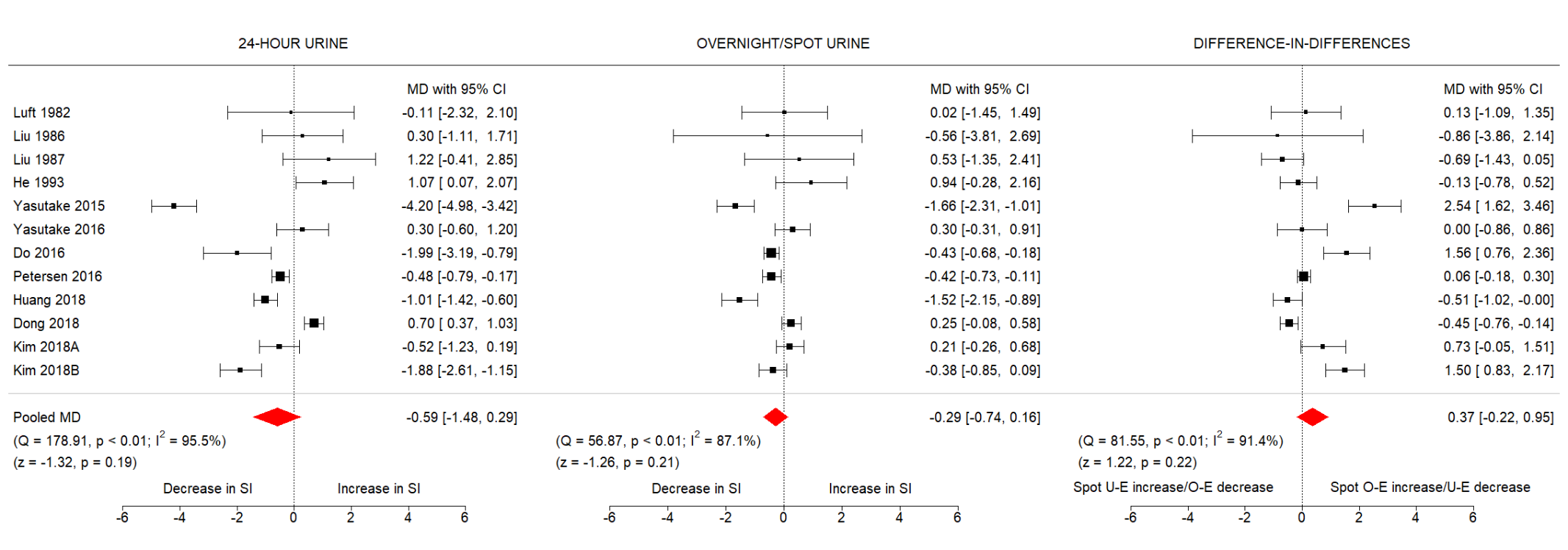


SI, salt intake; U-E, underestimated; O-E, overestimated.
